# More Is Not Always Better: Evaluation of 1D and 2D-LC-MS/MS Methods for Metaproteomics

**DOI:** 10.3389/fmicb.2019.00238

**Published:** 2019-02-14

**Authors:** Tjorven Hinzke, Angela Kouris, Rebecca-Ayme Hughes, Marc Strous, Manuel Kleiner

**Affiliations:** ^1^Department of Geoscience, University of Calgary, Calgary, AB, Canada; ^2^Institute of Pharmacy, Department of Pharmaceutical Biotechnology, University of Greifswald, Greifswald, Germany; ^3^Institute of Marine Biotechnology e.V., Greifswald, Germany; ^4^Department of Plant and Microbial Biology, North Carolina State University, Raleigh, NC, United States

**Keywords:** microbiota, microbiome, mock community, method evaluation, microbial ecology, Q Exactive, liquid chromatography, GeLC

## Abstract

Metaproteomics, the study of protein expression in microbial communities, is a versatile tool for environmental microbiology. Achieving sufficiently high metaproteome coverage to obtain a comprehensive picture of the activities and interactions in microbial communities is one of the current challenges in metaproteomics. An essential step to maximize the number of identified proteins is peptide separation via liquid chromatography (LC) prior to mass spectrometry (MS). Thorough optimization and comparison of LC methods for metaproteomics are, however, currently lacking. Here, we present an extensive development and test of different 1D and 2D-LC approaches for metaproteomic peptide separations. We used fully characterized mock community samples to evaluate metaproteomic approaches with very long analytical columns (50 and 75 cm) and long gradients (up to 12 h). We assessed a total of over 20 different 1D and 2D-LC approaches in terms of number of protein groups and unique peptides identified, peptide spectrum matches (PSMs) generated, the ability to detect proteins of low-abundance species, the effect of technical replicate runs on protein identifications and method reproducibility. We show here that, while 1D-LC approaches are faster and easier to set up and lead to more identifications per minute of runtime, 2D-LC approaches allow for a higher overall number of identifications with up to >10,000 protein groups identified. We also compared the 1D and 2D-LC approaches to a standard GeLC workflow, in which proteins are pre-fractionated via gel electrophoresis. This method yielded results comparable to the 2D-LC approaches, however with the drawback of a much increased sample preparation time. Based on our results, we provide recommendations on how to choose the best LC approach for metaproteomics experiments, depending on the study aims.

## Introduction

Metaproteomics, the analysis of expressed proteins in a microbial community (Wilmes and Bond, 2004), is a powerful tool which has enabled new insights into the role of microorganisms in a variety of environments. Metaproteomics not only provides information about gene expression of uncultured microorganisms, it can also be used for direct activity measurements with stable isotope probing (SIP) approaches (Jehmlich et al., 2016), the determination of carbon sources of individual species in microbial communities using stable isotope fingerprinting (protein-SIF) (Kleiner et al., 2018) and the assessment of community structure based on proteinaceous biomass (Kleiner et al., 2017). Numerous systems have been studied using metaproteomics, including acid mine drainage biofilms (Ram et al., 2005; Belnap et al., 2010), lichen (Schneider et al., 2011), marine symbioses (Markert et al., 2011; Gardebrecht et al., 2012; Kleiner et al., 2012; Wippler et al., 2016; Ponnudurai et al., 2017), plankton (Sowell et al., 2011), biogas plant communities (Heyer et al., 2016), and the human gut (Xiong et al., 2015a; Xiao et al., 2017). In the most common metaproteomic workflow, the protein mixture which is extracted from an environmental sample is digested into peptides. This peptide mixture is then analyzed in a mass spectrometer. There are numerous challenges associated with this workflow that must be addressed in order to obtain the largest possible number of identified and quantified proteins. The foremost challenge is high sample diversity and complexity. Others include finding a method for effective and unbiased cell lysis and protein extraction, generation of a database matching the sample, and correct assignment of peptides to proteins, especially for highly similar proteins from the same or different organisms (the protein inference problem) (Nesvizhskii and Aebersold, 2005; Muth et al., 2013; Wang et al., 2014; Xiong et al., 2015a; Kleiner et al., 2017). For two comprehensive reviews that provide more details on these challenges and how they can be addressed see VerBerkmoes et al. (2009) and Wilmes et al. (2015).

Here, we address the challenge posed by sample complexity, which translates into the large number of proteins or, ultimately, peptides that need to be analyzed. To increase the number of identifiable peptides and thereby proteins, it is crucial to reduce the sample complexity prior to measurement in the mass spectrometer (Taylor et al., 2009; Mostovenko et al., 2013; Weston et al., 2013). The sample complexity can be reduced by separating either proteins or peptides based on differing chemical properties. On-line separation is especially convenient, as the peptides are injected directly into the mass spectrometer after separation. In addition, on-line separations decrease bias and require less time, effort and material than manual off-line separations (Magdeldin et al., 2014; Camerini and Mauri, 2015; Richard et al., 2017). Frequently, one on-line peptide separation step is accomplished by reversed-phase liquid chromatography (RP-LC) of the peptides (Hsieh et al., 2013; Weston et al., 2013). To increase the separation – and thus the metaproteome coverage – an off-line sample separation can be used before the RP-LC step. The pre-separation of proteins by 1D SDS gel electrophoresis, or GeLC, is sometimes used in metaproteomics (Schneider et al., 2011; Stokke et al., 2012; König et al., 2016; Ponnudurai et al., 2017). Also possible is an off-line pre-separation of peptides (Keiblinger et al., 2012; Zheng et al., 2015). Alternatively to these off-line separations, a second on-line separation step can be used. For this additional on-line separation, a strong cation exchange (SCX) column can be added upstream of the RP column, resulting in 2D-LC separation (Taylor et al., 2009).

While multi-dimensional separation is currently the state-of-the-art approach for metaproteomics (Hettich et al., 2012), recent advances in column technology have made 1D-LC without additional separations a possible competitive alternative to multidimensional separations. For standard, single-organism proteomics it has been shown that longer columns enable extended 1D-LC runs, which achieve high resolution and at the same time use less sample material and take less MS runtime than multi-dimensional approaches (Thakur et al., 2011; Nagaraj et al., 2012; Pirmoradian et al., 2013).

One important difference between single-organism proteomics and multi-species metaproteomics is that, while in single-organism proteomics a fairly comprehensive set of peptides can be identified with a limited amount of MS run time, this is not the case in metaproteomics, where several orders of magnitude more peptides are present in a sample. This means that some proteomics methods cannot be immediately transferred to metaproteomics. For example, new methods such as BoxCar (Meier et al., 2018) have recently been developed, which allow to largely increase the number of quantified peptides in single-organism proteomics. This increased number of quantified peptides, however, can only be achieved by increasing the amount of time spent for the acquisition of MS spectra. Thus, less time is available for the MS/MS scans needed for peptide identification. BoxCar relies on the transfer of identifications between LC-MS/MS runs, as well as on spectral libraries to overcome the loss in MS/MS scans. Such a transfer of identifications between sample runs is, however, not feasible in metaproteomics, where each sample may contain different species and thus proteins. This makes the acquisition of maximal MS/MS scan numbers for peptide identifications indispensable in metaproteomics.

In this study, we compared the performance of 1D and on-line 2D-LC separation methods for metaproteomics. Additionally, we compared the performance of these on-line separations with the off-line GeLC method. We used a defined mock community, consisting of 32 strains and species, for this method comparison. We assessed the number of identified protein groups, unique peptides and peptide spectrum matches (PSMs), the detection of low-abundance species and the reproducibility of the methods. We show that, while 2D-LC methods can lead to more total identifications, they are not necessarily the best choice for metaproteomic studies. 1D-LC methods with long columns and gradients offer a suitable alternative in terms of throughput and ease of use. GeLC can be an alternative to 2D-LC, if no 2D-capable system is available. This data enables us to provide recommendations to researchers in the field of metaproteomics for the mass spectrometric experimental design based on their study goals.

## Materials and Methods

### Mock Community Generation

We used a defined mock community sample to test and compare different LC separation methods. The mock community corresponded to the UNEVEN community described by Kleiner et al. (2017). This mock community was assembled to cover a large taxonomic range and was not meant to resemble any specific metaproteome. Additionally, the aim was to address potential challenges for metaproteomic approaches, such as (1) large differences in species abundances in terms of proteinaceous biomass in the community, which challenges the dynamic range of the approaches, (2) different cell types influencing extraction efficiencies and (3) difficulties with protein inference due to highly similar strains from one species and multiple species from the same genus. The mock community contains a total of 32 different microbial species and strains of all three domains of life as well as viruses. The species and strains are present in different abundances in the UNEVEN community, similar to what can be expected for a natural microbial community. The community was assembled from pure cultures as described by Kleiner et al. (2017) and the detailed composition can be found in Supplementary Tables S3 and S6.

### Peptide Sample Preparation

#### LC-MS/MS (On-Line Separation)

Cells in mock community samples were disrupted by bead-beating (6.0 m/s, 45 s) in lysing matrix B tubes (MP Biomedicals) in SDT lysis buffer [4% (w/v) SDS, 100 mM Tris-HCl pH 7.6, 0.1 M DTT] followed by heating to 95°C for 10 min. Tryptic digests of protein extracts were prepared according to the filter-aided sample preparation (FASP) protocol described by Wiśniewski et al. (2009). Peptides were desalted with Sep-Pak C18 Plus Light Cartridges (Waters). Acetonitrile from the peptide elution step was exchanged for 0.1% formic acid (v/v) using a centrifugal vacuum concentrator. The desalting step was necessary to enable binding of peptides to the SCX column during sample loading for the 2D-LC methods. Peptide concentrations were determined using the Pierce Micro BCA assay (Thermo Scientific Pierce) according to manufacturer’s instructions. The peptide mixture was aliquoted and frozen at −80°C. For mass spectrometric analyses, fresh aliquots were regularly thawed and formic acid concentration increased to 0.2% (v/v). All aliquots used here were prepared at the same time from the same peptide mixture to eliminate between-sample variation.

#### GeLC-MS/MS (Gel-Based Separation)

To compare the on-line LC separation of peptides with the 1D pre-separation of proteins on an SDS gel, followed by 1D-LC separation (GeLC approach), aliquots of the UNEVEN mock community were prepared for GeLC as similarly as possible to the FASP approach above. Briefly, we disrupted cells with the same method as for the on-line separations, with the modification that no DTT was used in the lysis buffer. After centrifugation for 5 min at 21.000 × *g*, protein concentrations in the supernatant were determined using the Pierce BCA assay (Thermo Scientific Pierce) according to the manufacturer’s instructions for the enhanced protocol. 30 μg of protein were mixed with loading buffer (4x Laemmli Sample Buffer, Bio-Rad; containing 50 mM DTT) and separated on 12% polyacrylamide gels [12% (v/v) acrylamide, 0.38 M Tris pH 8.8, 0.1% (w/v) SDS, 0.1% (w/v) APS, 0.04% (v/v) TEMED]. Gels were fixed for 15 min [40% (v/v) ethanol, 10% (v/v) glacial acetic acid], washed twice in deionized water and stained with QC Colloidal Coomassie Stain (Bio-Rad) for 2 h. After overnight destaining, each gel lane was cut into 10 equal-sized pieces. In-gel digestion of proteins and peptide elution was done as described previously by Eymann et al. (2004) with several small modifications. In brief, gel pieces were washed three times (15 min, 900 rpm shaking, 37°C) in 300 μl destaining solution (200 mM ABC in 50% v/v acetonitrile) and dried for 30 min in a vacuum centrifuge. Gel pieces were rehydrated for 30 min in an aqueous 2 ng μl^−1^ trypsin solution (sequencing grade modified trypsin, Thermo Scientific Pierce). Trypsin solution not absorbed by the gel pieces was removed and the gel pieces were incubated at 37°C overnight for digestion. Peptides were eluted by adding 40 μl of distilled water and applying ultrasound for 15 min in a sonication bath. The supernatant was transferred to HPLC vials for analysis. For two biological replicates of the UNEVEN mock community (U1 and U2), peptides from two gel lanes were combined to increase loading amount.

### MS Analysis

#### 1D-LC-MS/MS and GeLC-MS/MS

For 1D-LC-MS/MS and GeLC-MS/MS analysis, an UltiMateTM 3000 RSLCnano Liquid Chromatograph (Thermo Fisher Scientific) with two 10-port valves in the column oven was used to load the respective amount of peptide mixture (Table 1) with loading solvent A (2% acetonitrile, 0.05% trifluoroacetic acid) onto a 5 mm, 300 μm ID C18 Acclaim^®^ PepMap100 pre-column (Thermo Fisher Scientific) at a flow rate of 5 μl min^−1^. The pre-column was then switched into line with the analytical column, which was either a 50 cm × 75 μm analytical EASY-Spray column packed with PepMap RSLC C18, 2 μm material (Thermo Fisher Scientific), heated to 45°C (1D and GeLC), or a 75 cm × 75 μm analytical column with the same packing material (Thermo Fisher Scientific), heated to 60°C (1D-LC only). An EASY-Spray source connected the analytical column to a Q Exactive Plus hybrid quadrupole-Orbitrap mass spectrometer (Thermo Fisher Scientific). Elution and separation of peptides on the analytical column was achieved at a flow rate of 225 nl min^−1^ using a gradient of eluent A (0.1% formic acid) and eluent B (0.1% formic acid, 80% acetonitrile) with the times as specified in Supplementary Table S1. Eluting peptides were ionized using electrospray ionization. Two wash runs with injection of 20 μl acetonitrile and 99% eluent B and one blank run were done between samples to reduce carryover. Data was acquired in the Q Exactive Plus as described by Petersen et al. (2016).

**Table 1:** Overview of LC methods developed and tested in this study.

Method	Method overview^a^	Peptide loaded [μg]	Runtime [h]^b^
**2D-LC (all with 50 cm analytical column)**			
2D|3salt_4.5_	bt; 5, 100, 2000 mM (each 300 min)	4.5	20
2D|3salt_9_	bt; 5, 100, 2000 mM (each 300 min)	9	20
2D|11salt_4.5_	bt; 1, 2, 5, 10, 20, 50, 100, 200, 500, 1000, 2000 mM (each 120 min)	4.5	24
2D|11salt_9_	bt; 1, 2, 5, 10, 20, 50, 100, 200, 500, 1000, 2000 mM (each 120 min)	9	24
2D|10pH_G1	bt; pH 2.5, 3.0, 3.5, 4.0, 4.5, 5.0, 5.5, 6.0, 7.0, 8.0 (each 120 min)	4.5	22
2D|10pH_G2_4.5_	bt (300 min); pH 2.5, 3.0, 3.5, 4.0, 4.5, 5.0, 5.5, 6.0, 7.0, 8.0 (each 120 min)	4.5	25
2D|10pH_G2_9_	bt (300 min); pH 2.5, 3.0, 3.5, 4.0, 4.5, 5.0, 5.5, 6.0, 7.0, 8.0 (each 120 min)	9	25
2D|5pH+2salt	bt (228 min); 1 mM (228 min); pH 3.0 (120 min), 4.0 (105 min), 4.5 (90 min), 5.0 (90 min), 6.0 (79 min); 2000 mM (60 min)	4.5	16.7
2D|6pH+1salt_G1	bt (228 min); pH 2.5 (228 min), 3.5 (120 min), 4.5 (90 min), 5.0 (90 min), 6.0 (79 min), 8.0 (60 min); 2000 mM (60 min)	4.5	15.9
2D|6pH+1salt_G2	bt (466 min); pH 2.5 (228 min), 3.5 (120 min), 4.5 (90 min), 5.0 (90 min), 6.0 (79 min), 8.0 (60 min); 2000 mM (60 min)	4.5	19.9
2D|8pH+1salt_G1_4.5_	bt (300 min); pH 2.5 (120 min), 3.0 (120 min), 3.5 (120 min), 4.0 (105 min), 4.5 (105 min), 5.0 (105 min), 6.0 (105 min), 8.0 (80 min); 2000 mM (80 min)	4.5	20.7
2D|8pH+1salt_G1_9_	bt (300 min); pH 2.5 (120 min), 3.0 (120 min), 3.5 (120 min), 4.0 (105 min), 4.5 (105 min), 5.0 (105 min), 6.0 (105 min), 8.0 (80 min); 2000 mM (80 min)	9	20.7
2D|8pH+1salt_G2	bt (228 min); pH 2.5 (228 min), 3.0 (120 min), 3.5 (120 min), 4.0 (105 min), 4.5 (90 min), 5.0 (90 min), 6.0 (79 min), 8.0 (60 min); 2000 mM (60 min)	4.5	19.7
2D|8pH+4salt_4.5_	bt (300 min); 1 mM (120 min); pH 2.5 (120 min), 3.0 (120 min), 3.5 (120 min), 4.0 (105 min), 4.5 (105 min), 5.0 (105 min), 6.0 (105 min), 8.0 (80 min); 500 mM (80 min), 1000 mM (80 min), 2000 mM (80 min)	4.5	25.3
2D|8pH+4salt_9_	bt (300 min); 1 mM (120 min); pH 2.5 (120 min), 3.0 (120 min), 3.5 (120 min), 4.0 (105 min), 4.5 (105 min), 5.0 (105 min), 6.0 (105 min), 8.0 (80 min); 500 mM (80 min); 1000 mM (80 min); 2000 mM (80 min)	9	25.3
2D|10pH+1salt	bt (228 min); pH 2.5 (228 min), 3.0 (120 min), 3.5 (120 min), 4.0 (105 min), 4.5 (90 min), 5.0 (90 min), 5.5 (90 min), 6.0 (79 min), 7.0 (79 min), 8.0 (60 min), 2000 mM (60 min)	4.5	22.5
2D|11salt^c^	bt; 1, 2, 5, 10, 20, 50, 100, 200, 500, 1000, 2000 mM (each 120 min)	11	24
**1D-LC**			
**50 cm analytical column**			
1D|8h_50_1.6_	460min_Pre20 (460 min plus 20 min pre-equilibration)	1.6	8
1D|8h_50_2_	460min_Pre20 (460 min plus 20 min pre-equilibration)	2	8
1D|8h_50_2.5_	460min_Pre30 (460 min plus 30 min pre-equilibration to also work with the 75 cm column)	2.5	8.2
1D|12h_50	720min_Pre30 (720 min plus 30 min pre-equilibration to also work with the 75 cm column)	2.5	12.5
1D|4h^c^	260min_Pre20 (260 min plus 20 min pre-equilibration)	0.8–2	4.7
1D|8h^c^	460min_Pre20 (460 min plus 20 min pre-equilibration)	2	8
**75 cm analytical column**			
1D|8h_75	460min_Pre30 (460 min plus 30 min pre-equilibration)	2.5	8.2
1D|12h_75	720min_Pre30 (720 min plus 30 min pre-equilibration)	2.5	12.5
**GeLC (50 cm analytical column)**			
GeLC	10x (Load, 90 min)	(30–60)^d^	16.7

*^a^ For description of the gradients, see Supplementary Table S1. bt, breakthrough; i.e., peptides that do not sufficiently bind to the SCX column and are directly eluted onto the C18 RP column before any salt or pH steps. Concentrations correspond to NaCl concentrations in loading buffer B. pH values correspond to commercial pH plugs (CTIBiphase buffers, Column Technology, Inc.). ^*b*^Method runtime without intermediary times for washing etc., but with pre-equilibration for 1D methods. ^*c*^This method was run on four biological replicates as described by Kleiner et al. (2017). ^*d*^Protein concentration was determined before loading samples on the gel.*

#### 2D-LC-MS/MS

For the 2D-LC-MS/MS experiments, the same LC as for the 1D experiments was used. The respective amount of peptide mixture (Table 1) was loaded with loading solvent B (2% acetonitrile, 0.5% formic acid) onto a 10 cm, 300 μm ID Poros 10 S SCX column (Thermo Fisher Scientific) at a flow rate of 5 μl min^−1^. The specific plumbing scheme used in the RSLCnano corresponded to the standard set up recommended by the manufacturer for on-line 2D salt plug separations^1^. During loading, the C18 pre-column (see above) was in-line downstream of the SCX column to capture peptides that did not bind to the SCX column (breakthrough). After loading, the C18 pre-column was switched in-line with the 50 cm × 75 μm analytical column (same as for 1D) and the breakthrough was separated using a gradient of eluent A and eluent B (Supplementary Table S1). Subsequently, elution of peptides from the SCX to the C18 pre-column (same as for 1D-LC) took place by injection of 20 μl of salt plugs (different concentrations of NaCl in loading buffer B) or pH plugs (CTIBiphase buffers, Column Technology, Inc.) from the autosampler. The salt concentrations and pH steps were dependent on the method used (Table 1). The C18 pre-column was then again switched in-line with the analytical column and peptides separated with gradients of eluent A and B (Supplementary Table S1). Two washes of the SCX column (injection of 20 μl 4 M NaCl in loading solvent B, 100% eluent B), one RP column wash (injection of 20 μl acetonitrile, 99% eluent B) and one blank run were done between samples to reduce carryover. Data acquisition in the mass spectrometer was done as described by Petersen et al. (2016).

### Protein Identification and Quantification

The same database as in Kleiner et al. (2017) was used, containing all protein sequences from the reference genomes of the organisms present in the mock community (Supplementary Tables S3, S6). The database also included the cRAP protein sequence database with common laboratory contaminants^2^. The database contained 123,100 protein sequences and is available from the PRIDE (Vizcaíno et al., 2016) repository (PXD008017).

The raw MS files were searched against this database using the MaxQuant software version 1.5.8.3 (Cox and Mann, 2008; Tyanova et al., 2016). MaxQuant identifies protein groups, which are defined as the set of all proteins that cannot be separated based on their detected peptides (Cox and Mann, 2008). At least one unique peptide was required for protein group identification, in addition to an FDR of 1% on the peptide and on the protein group level. All other parameters were left at default values.

For MaxQuant analyses of several runs together, the “Second peptides” and the “Match between runs” options were disabled. Where needed, we used the normalized spectral abundance factor (NSAF) (Zybailov et al., 2006) for quantification. To quantify the number of proteins identified per species and to calculate protein isoelectric point (pI) distributions, size distributions and NSAFs, we used the first protein in the Majority Protein ID assignment, if different proteins were present in the protein group.

### Data Evaluation

For data evaluation, the proteinGroups.txt output from the MaxQuant searches was filtered as follows: protein groups with zero MS/MS (i.e., only identified by co-eluting ‘second peptides’), or those identified by modification site only as well as reverse hits were removed. We exclusively defined protein groups which passed these criteria as being identified and used them for further analyses. Missing values were replaced by zero for calculations. For overlap calculations, a custom Venn diagram tool from the University of Gent was used^3^. Protein pIs were calculated using the package seqinR (Charif and Lobry, 2007) in R (R Core Team, 2018).

## Results and Discussion

### Method Development

We tested different 1D and 2D-LC separation methods to find which ones are the best for metaproteomics. For the 1D-LC separations, we used C18 RP-LC with acetonitrile-based gradients. For 2D-LC, we used SCX LC with step-wise salt (Taylor et al., 2009) or pH bumps (Dai et al., 2005) as the first dimension and C18 RP-LC as the second dimension. The salt and pH bumps were injected directly from the autosampler, similar to what has been described by Taylor et al. (2009). Our system had separate SCX and C18 pre-columns and a switching valve that connected the C18 pre-column to the analytical column, while Taylor et al. (2009) used a biphasic pre-column connected to the analytical column in a vented-column setup.

We compared different gradient lengths and shapes for both 1D and 2D-LC as well as combinations of salt and pH steps for the 2D-LC (Table 1). We chose the different gradient and salt/pH bumps to achieve a roughly equal distribution of eluting peptides over the gradients and between steps.

Additionally, we compared the on-line 1D and 2D-separations to a GeLC approach, for which we used 12% SDS gels and 1D C18 RP-LC.

### How to Get the Most Proteins?

**FIGURE 1:**
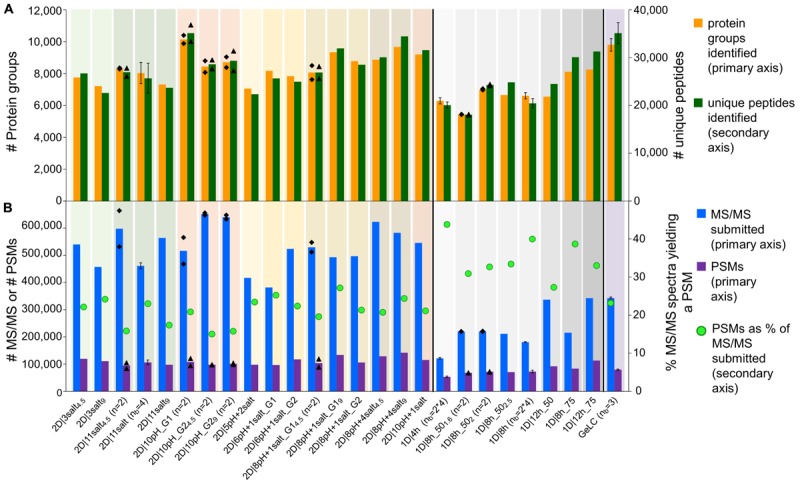
Comparison of data amount obtained with different 1D and 2D-LC-MS/MS and the GeLC method. **(A)** Number of protein groups and unique peptides (secondary axis) identified by MaxQuant. **(B)** Number of submitted MS/MS, PSMs and the percentage of PSMs of submitted MS/MS (secondary axis). For *n* = 2, the means of two independent runs with the same method (bars) and the two individual values (black diamonds) are shown. For *n*_b_ = 3, *n*_b_ = 4 and *n*_b_ = 2^∗^4, the means of three (*n*_b_ = 3) or four biological replicates (*n*_b_ = 4), or four biological replicates, measured in technical duplicate (*n*_b_ = 2^∗^4), are shown. Error bars indicate standard deviations.

We achieved the highest number of identified protein groups [i.e., sets of proteins which cannot be separated based on their peptides by MaxQuant (Cox and Mann, 2008)] with a 2D-LC method, 2D|10pH_G1 (Figure 1A). This method had 10 pH plugs, each eluted with a 120 min gradient. With 2D|10pH_G1 we identified a mean of 10,148 protein groups, based on 35,127 unique peptides.

Interestingly, two of the 1D methods performed better than several 2D methods. With the best 1D method (1D|12h_75) we identified 8,261 protein groups, corresponding to 31,283 unique peptides. With a 75 cm column, we increased the number of identifications for an 8 h as well as for a 12 h 1D run as compared to the 50 cm column (Supplementary Table S2).

The total number of identified unique peptides correlated well with the number of protein groups for all methods (Figure 1A). However, some methods provided more unique peptides per protein group than others. The best method in this regard was 1D|12h_75 with 3.8 unique peptides/protein group (Supplementary Table S2). This higher number of unique peptides per protein group increases the identification and quantification confidence of the protein groups identified in 1D|12h_75 as compared to the other methods (Mallick and Kuster, 2010).

The percentage of protein groups with one unique peptide and one peptide total (i.e., the unique peptide is the only peptide found) and only one PSM was mostly higher for the 2D methods (Supplementary Table S2), likely because in the 2D methods peptides of less abundant proteins were also targeted for identification (in addition to higher-abundant ones detected by 1D methods as well) and these peptides of low-abundant proteins have a lower likelihood of being detected. The datasets for which four biological replicates were measured generally had a higher proportion of protein groups with one (unique) peptide or one PSM, which might be due to the additional variance introduced by biological replication.

In terms of time efficiency, i.e., protein groups identified per minute of gradient runtime (see also Köcher et al., 2011), the 1D|4h method outperformed every other method, with 22.5 protein groups identified per min. In comparison, the best 2D method in this regard (2D|6pH+1salt_G1) only reached about 8.6 protein groups per min and the second best 1D method (1D|8h_75) identified 16.6 proteins per min (Supplementary Table S2).

We also tested the effect of loading different amounts of digested sample on the identification numbers. The sample amount has to be high enough to allow for sufficient ion intensities for peptide identifications, while not so high as to overload the column and cause ion suppression. The amount of sample needed thus depends on the run length: longer runs need more sample to achieve high enough ion intensities over the whole run. We did see the different effects of loading not enough and loading too much sample: in some cases, the number of identified protein groups increased when we used more sample material (especially when we loaded 2 μg instead of 1.6 μg as measured by BCA assay for the 1D|8h_50 methods). For some methods, we identified fewer protein groups after loading more material. While 2 μg of peptide are sufficient for a 1D 8 h run, for 2D runs at least 4 μg should be used, as we still see an increase in identifications for 9 μg as compared to 4 μg for several 2D methods.

Overall, we observed good performance of 1D-LC separation approaches with a long gradient and long analytical column for metaproteomics. When comparing the number of identified protein groups between the 4 h 1D method and the 8 h 1D method run on the same column length (50 cm) and with at least 2 μg peptide loaded for the 8 h runs, we found that the 4 and 8 h runs both performed equally. The 4 h method only identified on average 6.2% less protein groups as compared to the 8 h methods (Supplementary Figure S1 and Supplementary Table S2).

In the following, we will compare the data obtained in this study with existing literature data. We would like to add a word of caution though, since to our knowledge LC-MS/MS methods for metaproteomics have never been assessed in this depth before and both LC instruments and mass spectrometers have undergone major improvements over the last few years. Comparisons with numbers reported in the literature can thus only give a very rough impression of the comparative method performance and must be interpreted with care. Similar to our study, where we identified up to over 8,000 proteins with 1D runs using 50 and 75 cm columns, other authors reported good performance of 1D methods using pure cultures or simpler communities. Nagaraj et al. (2012) identified, on average, over 3,900 yeast proteins by using a 4 h LC gradient with a 50 cm column. Richards et al. (2015) describe a method to identify up to 4,000 yeast proteins in 70 min using a 30 cm column. Likewise, Pirmoradian et al. (2013) detected over 4,800 protein groups in a human cell line with a 4 h run and a 50 cm column. In bacterial symbiont-containing gill tissue of a lucinid clam, Petersen et al. (2016) identified up to 1,400 proteins of the symbiont alone using a 50 cm column and a 460 min gradient. Another study of symbiont-containing mussel gills with the same column and gradient lengths led to over 7,700 identified proteins of host and symbionts (Rubin-Blum et al., 2017).

In contrast to our best 2D runs, which resulted in over 10,000 identified proteins, numbers reported in the literature for metaproteomes analyzed with 2D setups are lower. For example, a 2D-LC analysis of a symbiosis consisting of a marine oligochaete with at least four different bacterial species yielded a total of 4,355 protein groups (Wippler et al., 2016). In fermented fish microbiota, a total of 2,175 proteins were identified from different subsamples after 2D-LC (Ji et al., 2017). Two studies of human infant gut microbiota used 2D-LC to identify up to 1,264 protein groups (Xiong et al., 2015b) or up to 4,031 proteins (Young et al., 2015).

### More MS/MS – More Identifications?

The number of MS/MS spectra and the number of PSMs generated from these MS/MS spectra are important parameters to assess metaproteomics data quality: the identification of protein groups is based on the MS/MS spectra that match to peptides in a database comparison and thus generate PSMs (Zhang et al., 2013). Besides being the building blocks of protein identifications, PSMs can also be used to quantify proteins via spectral counting based methods, e.g., with NSAFs (Zybailov et al., 2006).

The matching of MS/MS spectra to peptides in the database depends on the quality of the database, i.e., how well the database fits to the actual sample (Timmins-Schiffman et al., 2017), and on the quality of the MS/MS spectra themselves. As we used a mock community with a known composition of sequenced organisms, we were able to remove confounding effects of database quality. We however want to emphasize the importance of using a database that actually fits the sample in question.

The 1D-LC methods generally produced less MS/MS spectra than the 2D-LC methods, due to the shorter time available to the mass spectrometer to acquire spectra in the 1D runs (Hsieh et al., 2013). The proportion of identified MS/MS spectra (PSMs), on the other hand, was higher for the 1D runs than for the 2D runs (Figure 1B and Supplementary Table S2). The likely reason for this is that in the 2D runs not only the higher abundant peptides were sampled (as during the 1D runs), but also more spectra of low abundant peptides were acquired during 2D runs due to the better separation, as indicated by more identified proteins of low-abundance species in 2D runs. Those low-abundance peptides also include non-tryptic peptides which cannot be identified with the search engine settings used, leading to a lower proportion of identified MS/MS spectra in 2D runs, while the absolute number of PSMs is still higher in 2D runs, e.g., twice as high in the 2D|10pH runs as compared to a 4 h 1D run (Supplementary Figure S1).

We also evaluated our LC methods in terms of absolute number of PSMs (Figure 1B). The total number of PSMs is generally higher for 2D than for 1D-LC methods, with the exception of 1D|12h_75. Interestingly, 2D|10pH_G1, which identified the most protein groups and unique peptides, led to a comparatively low number of about 108,000 PSMs. This indicates the higher separation efficiency of the 2D|10pH_G1 method, as new peptides are targeted more often for sequencing than in the other 2D methods.

### How Beneficial Are Run Repetitions?

**FIGURE 2:**
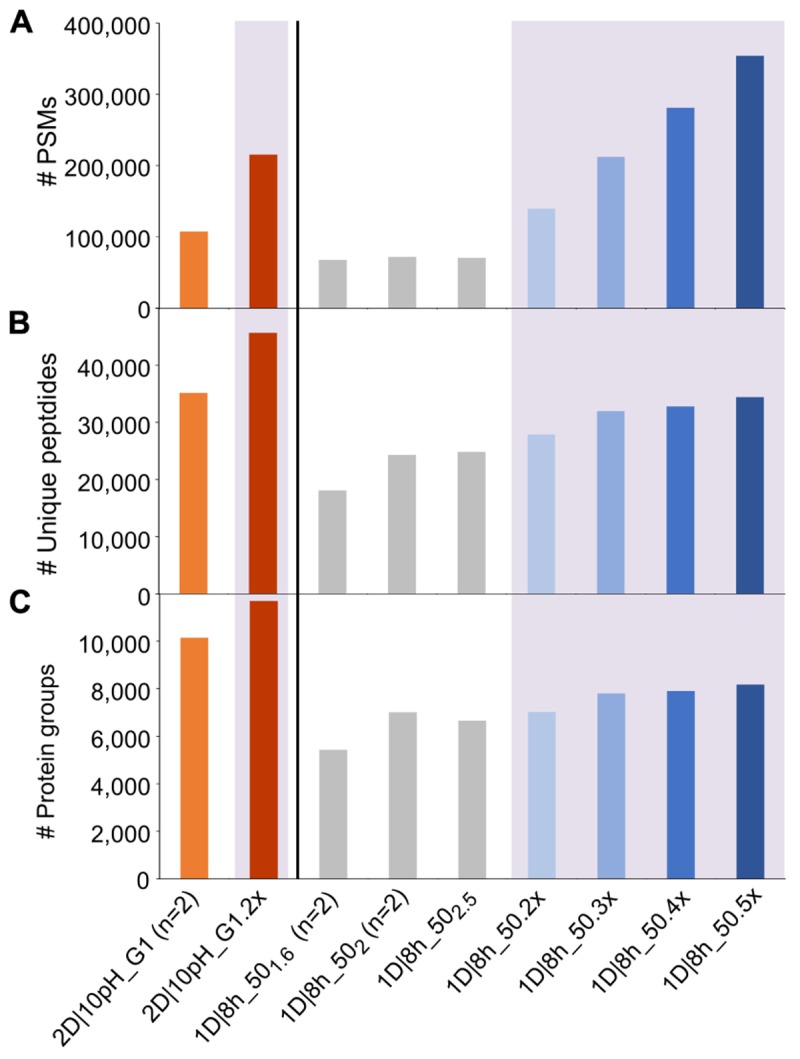
Number of PSMs, unique peptides and protein groups for replicate runs of 2D|10pH_G1 and repetitions of 1D|8h_50 runs. **(A)** Number of PSMs, **(B)** number of identified unique peptides, **(C)** number of identified protein groups. For (*n* = 2), the mean value of two independent runs is shown. Results for searches combining runs have a purple colored background and are labeled with the number of runs searched together in MaxQuant, followed by “x” (e.g., 2D|10pH_G1 (*n* = 2) means that two runs have been searched separately and the mean is shown, whereas 2D|10pH_G1.2x means that both replicates have been run in MaxQuant as one experiment and the output of the combined search is depicted).

One potential way to increase metaproteome coverage is to repeat a run once or multiple times. In order to evaluate the effect of repeating runs, we used increasing numbers of the 1D|8h_50 runs as one experiment in MaxQuant searches. Additionally, we compared the effect of running our best 2D method in terms of protein groups identified (2D|10pH_G1) once versus in technical replicate (Figure 2 and Supplementary Table S3).

Each additional run of the 1D|8h_50 runs increased the number of PSMs. The total number of unique peptide and protein group identifications also increased, albeit not as much as the number of PSMs. Similarly, the replicate run 2D|10pH_G1.2x led to double the amount of PSMs compared to a single run, while the number of unique peptides increased by 29.9%, and the number of identified protein groups by 15.3%. The percentage of protein groups with one unique peptide, one peptide total or one PSM total decreased with more runs (Supplementary Table S3). Overall, there is a decreasing benefit of increasing run numbers for the protein group identifications, which has also been observed by others (Thakur et al., 2011). The reason for this is that during replicate runs, foremost peptides of the same (more abundant) protein groups are sequenced. Therefore, run repetition increases the amount of spectra for proteins already identified in the first run and, to a lesser extent, metaproteome coverage.

We also analyzed how a single long 2D run competes against repeated shorter 1D runs. While in three combined runs of 1D|8h_50 we identified about 7,800 protein groups, the 2D|10pH_G1 alone led to over 10,000 protein groups, even though three 1D|8h_50 runs together have about the same total runtime as a single 2D|10pH_G1 run (24 vs. 22 h). Köcher et al. (2011), too, identified more protein groups in HeLa digests with a single long run than with replicate shorter runs, using 1D-LC with a long analytical column. The number of PSMs, on the other hand, was almost double for the three 1D|8h_50 runs (over 212,000 as compared to about 108,000 for 2D|10pH_G1). Thus, while the 2D|10pH_G1 led to more protein groups overall, the 1D|8h_50 runs taken together contained on average more information per protein.

### How Reproducible Are the LC Methods?

We assessed the reproducibility of selected methods in terms of total per species protein quantification within and between methods and overlap of identified protein groups within methods. For this, we used the biological and technical replicate runs of the UNEVEN mock community from Kleiner et al. (2017). In this study, 1D methods (1D|4h, 1D|8h) and a 2D method (2D|11salt) were used to measure four independently generated biological replicates of the UNEVEN mock community. In addition to this, for the 1D methods all samples were measured in technical duplicates.

**FIGURE 3:**
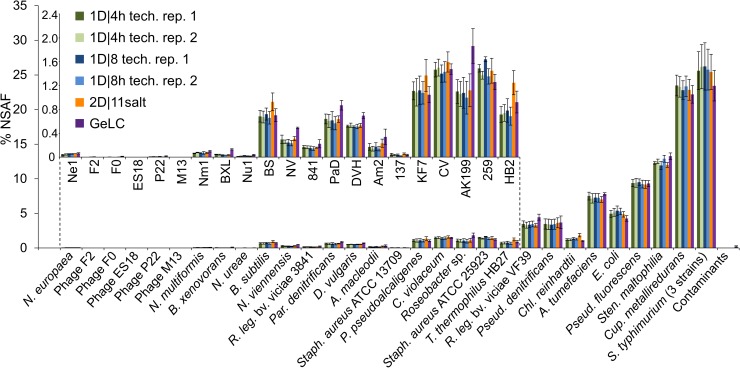
% NSAFs summed up for each organism in the mock community. Each bar represents the mean of four biological replicates analyzed with 1D-LC or 2D-LC and three biological replicates analyzed with GeLC-MS/MS. For the 1D-LC-MS/MS methods, technical replicate (tech. rep.) datasets are presented as separate bars. Error bars indicate standard deviations. Small insert: % NSAF for the species with lower abundance. Data for this figure in tabular format can be found in Supplementary Table S4.

To assess how reproducible the methods are in terms of quantification of individual species’ proteins, we summed the relative abundance of proteins of each species for all runs (Figure 3). We found that the mean and standard deviations of biological replicates are comparable between technical replicates of the same method and between different methods.

Additionally, we compared the method reproducibility between technical and biological replicates for the total metaproteome coverage. For this, we calculated the overlap between identified protein groups of technical and biological replicates (Supplementary Table S5). Technical replicates of the 1D methods had an overlap between 82 and 86%. These values are similar to previously published overlap values for technical replicates in proteomic experiments with yeast (i.e., a less complex sample than our mock community): Richards et al. (2015) reported an 83% overlap of protein identifications in five technical replicates of 1 h 1D runs. Nagaraj et al. (2012) noted a 92% overlap of six technical replicates of 4 h 1D runs on the protein level. While Nagaraj et al. (2012) enabled the “Match between runs” option in MaxQuant, which should increase the overlap between runs, we deliberately disabled this option such that only peptides identified via MS/MS sequencing during a run in our analysis were included.

On average between 74 and 76% of the protein groups identified in one run were detected in all biological replicates, regardless of the method (1D or 2D) used (Supplementary Table S5). A lower overlap between biological replicates as compared to technical replicates is to be expected, since biological replication introduces more variation. Moreover, two technical replicates, but four biological replicates (each of which represents an additional source of variance) were used. In summary, in terms of reproducibility, 1D and 2D methods perform comparably.

### How to Acquire Data for Low-Abundance Organisms?

An important aspect of metaproteomics is the analysis depth – how do we identify proteins of as many community members as possible, including low-abundance ones, that despite their low abundance can be important members of the system (Podar et al., 2007; Hajishengallis et al., 2011; Baldrian et al., 2012)?

**FIGURE 4:**
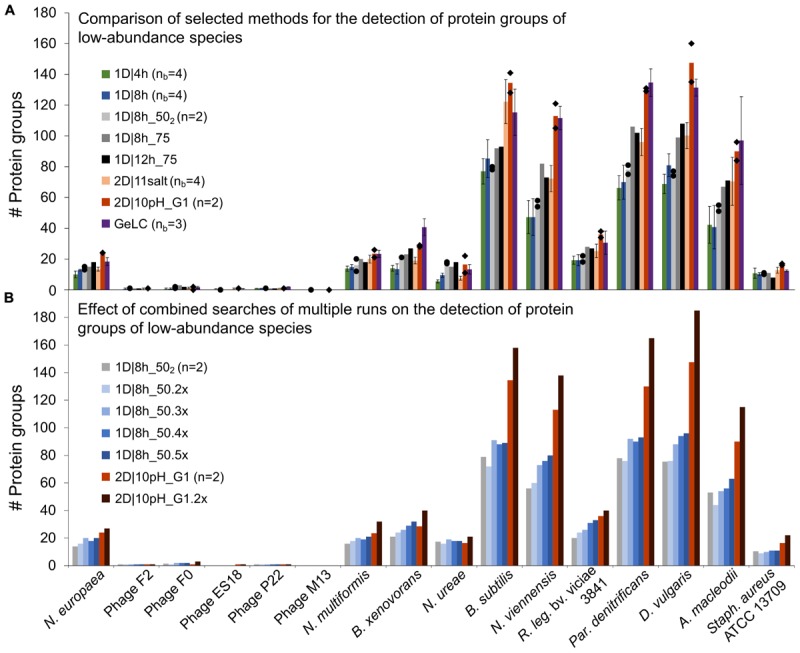
Number of protein groups of low-abundance organisms in the mock community identified by different LC methods. **(A)** Comparison of selected 1D and 2D-LC methods and the GeLC method. Shown are means (bars), individual values (black diamonds) for *n* = 2 and standard deviations for *n*_b_ = 3 and *n*_b_ = 4. **(B)** Results for searches combining multiple runs. The number of runs combined for searches in MaxQuant is indicated by the number of runs followed by “x.” n, number of technical replicates; n_b_, number of biological replicates.

To determine which LC method is best suited to detect low-abundance organisms, we analyzed the datasets for which four biological replicates were measured (1D|4h, 1D|8h, and 2D|11salt), as well as the 1D|8h_50_2_, 1D|8h_75, 1D|12h_75, and 2D|10pH runs. We especially considered the 16 organisms that had <1% total protein abundance in the mock community (Figure 4A and Supplementary Table S6 for all organisms).

Surprisingly, the 1D|4h and 1D|8h_50 methods performed similarly in detecting proteins of low-abundance species. For several organisms, the 2D|10pH_G1 method clearly outperformed the other methods. For the viruses (F2, F0, ES18, P22, and M13), which consist of only very few major proteins, there was almost no difference between methods. Aside from the viruses, which have a very small genome, we did not see a correlation between genome size (which roughly translates into the theoretically observable number of proteins) and number of proteins identified (Supplementary Tables S3, S6).

For the lowest-abundance bacterium (*Nitrosomonas europaea*, 0.082% total protein abundance in the mock community), at least eight protein groups were detected by every run considered and with 2D|10pH_G1, we detected 24 protein groups containing this organism. The protein groups which contain only *N. europaea* proteins, for example, housekeeping proteins such as a 50S ribosomal protein (Ne1_Q82VV4), allow us to confidently deduce the presence of this species in the sample. Additionally, some of the detected proteins such as ammonia monooxygenase (Ne1_Q04508), which was identified even in 1D|4h runs, would, when analyzing an uncharacterized community, provide a hint that this organism is an ammonia-oxidizing chemoautotroph. Nitrosocyanin (Ne1_Q820S6) and cytochrome c-552 (Ne1_P95339) were also identified already in several 1D|4h as well as in at least one 2D|10pH_G1 run. Nitrosocyanin is a red copper protein with a potential central role in metabolism of ammonia-oxidizing bacteria, while cytochrome c-552 is part of the electron transport chain (Arciero et al., 2002; Zorz et al., 2018). In both 2D|10pH_G1 runs, phosphoenolpyruvate synthase (Ne1_Q81ZR7), phosphoenolpyruvate carboxylase (Ne1_Q82WS3) and phosphoglycerate kinase (Ne1_Q82XE8), all involved in carbohydrate metabolism, were also identified.

Besides more peptide separation, run replication could also increase the data from low-abundance community members. To test whether run repetitions could indeed lead to higher analysis depth, we analyzed repeated runs of 1D|8h_50 and 2D|10pH_G1 (Figure 4B and Supplementary Table S3). The number of identified protein groups increased for most organisms with more runs. For the viruses there was very little-to-no change in protein identification numbers, again likely due to the limited total number of abundant proteins in these organisms.

A single 2D|10pH_G1 run mostly provides more protein groups for low-abundance organisms than three repetitions of a 1D|8h_50 run, while both have roughly the same runtime (22 vs. 24 h). Thus, for detecting low-abundance community members (or low-abundance proteins) a 2D-LC separation is better suited than repeated 1D-LC runs. Since low-abundance proteins are generally less reliably identified than high-abundance ones (Zubarev, 2013), replicate 2D runs would be even better to generate more data from low-abundance proteins or species, if those are important for the study question.

### How Do the On-Line LC-MS/MS Approaches Compare to the GeLC-MS/MS Approach?

We compared the performance of a standard GeLC approach, which has been used in different metaproteomic studies (Schneider et al., 2011; Stokke et al., 2012; König et al., 2016; Ponnudurai et al., 2017), to the on-line-only approaches that we tested. The total number of protein groups and unique peptide identifications was comparable between the GeLC approach and our best performing 2D-LC-MS/MS approach (2D|10pH_G1) and thus higher than our 1D-LC-MS/MS-only approaches. A similar performance difference was recently observed in a study of biogas plant communities, where a GeLC method performed much better than a 120 min 1D-LC-MS/MS method (Wenzel et al., 2018). The GeLC method also performed comparably to the 2D methods in terms of run reproducibility (Figure 3) and identification of protein groups of low-abundance organisms (Figure 4).

The number of MS/MS generated by the GeLC approach was in the range of the 1D|12h runs. In contrast, the % identified spectra was much lower than in the 1D approaches and comparable to that of the 2D methods (Figure 1). Taken together, this indicates that the GeLC approach leads to the acquisition of a lower number of “redundant” spectra from the same peptides, as shown by the highest unique peptides to PSM ratio among all tested methods.

One potential concern with GeLC approaches is that no SDS gel electrophoresis method exists that works equally well for all protein sizes (Rabilloud et al., 2009). Therefore, it can be expected that biases, especially concerning very small and large proteins, are introduced by this step. Considering this, we checked our data for size and, additionally, pI biases in the identified proteins. We noted a small bias of the GeLC method against smaller proteins and proteins with a very low or high pI (Supplementary Table S7). For example, whereas in all on-line approaches proteins with a length of <150 amino acids made up 15–17% of all proteins, in the GeLC identifications this protein size fraction only made up 11.7%.

While 2D on-line separations and the GeLC approach overall performed equally well, the amount of sample preparation work and the possible throughput is not comparable between the methods: with the FASP approach, peptides for analysis can be generated in 1–1.5 days, and hands-on time is mainly limited to adding reagents to the filter units. For the GeLC approach, at least 2.5 days are necessary, with substantial hands-on time during gel slicing and washing. Even more important is the fact that there is a large difference in the number of samples that can be prepared in parallel. With FASP, which is used for preparation of peptides for the on-line only approaches, up to 44 samples can be prepared in parallel with a high-capacity centrifuge rotor. The number of reaction tubes that fit into the rotor is the main limitation for parallelization during FASP preparations. During the GeLC approach, on the other hand, each sample is split in at least 10 subsamples, meaning that preparing more than 3–4 samples in parallel is hardly feasible. This translates into an at least 10-fold higher sample preparation throughput for FASP with subsequent on-line separation than for the GeLC-MS/MS approach.

## Conclusion

In this study, we tested 1D and 2D-LC methods for metaproteomics. We included different gradient lengths, peptide loads, analytical column lengths (50 and 75 cm), 2D separations (salt and pH bumps), numbers of run repetitions, as well as reproducibility estimates with biological and technical replicates. We also compared these on-line separation methods with the off-line GeLC method. A graphical summary of our main findings can be found in Supplementary Figure S1.

We demonstrated that, when using a 50 cm column, an increase from 4 to 8 h 1D run time leads to only a small gain in identified protein groups, whereas a further increase in run length to 12 h does not improve the number of identified protein groups. This makes the 1D 4 h run the most time-effective choice (Figure 1 and Supplementary Figure S1).

Peptide load has a strong influence on the number of identified protein groups: up to a certain threshold, loading more peptide increases the number of identifications, after which the number of identifications decreases again (Figure 1).

When switching from a 50 cm to a 75 cm column for 8 and 12 h 1D runs, we found a large gain in identification numbers (Figure 1 and Supplementary Figure S1).

For the 2D runs, we found the best performance for the method using 10 pH bumps, each with a RP runtime of 120 min. This method outperforms all other methods for total number of identified protein groups (Figure 1) and the detection of protein groups of low-abundance species (Figure 4).

Repeating runs several times does not lead to a large gain in metaproteome coverage, but rather increases the data amount for already identified proteins (Figure 2).

When comparing the reproducibility of 1D versus 2D LC runs of biological replicate samples and technical replicate runs, we found that both approaches perform equally well (Figure 3 and Supplementary Table S5).

The GeLC approach performs equally well as the 2D method with 10 pH bumps (Figure 1), but is extremely limited in throughput as compared to the on-line methods.

**FIGURE 5:**
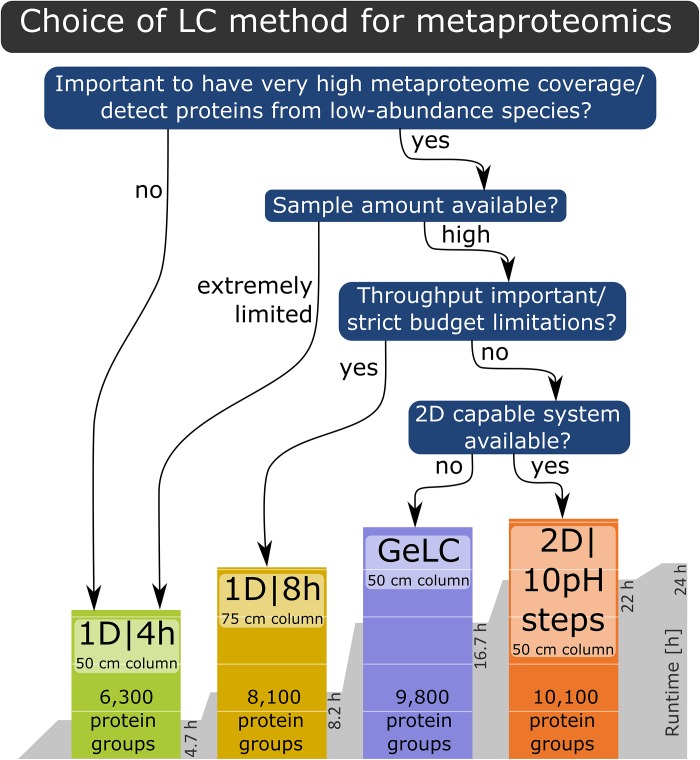
Decision tree for the LC method to choose for a specific experiment, based on the results of our study.

Based on our findings, we provide the following recommendations for LC method selection (Figure 5): if the goal is a very high metaproteome coverage or the detection of proteins from low-abundance species, then either a 1D|8h gradient with a 75 cm column, the GeLC approach or a 2D LC run should be used, depending on restrictions of sample quantity, equipment availability and throughput as well as budget considerations. If the sample amount is extremely limited or the characterization of proteins from low-abundance community members is not the primary goal, then a 1D|4h run with a 50 cm column is well-suited for metaproteomics experiments.

## Data Availability

The mass spectrometry proteomics data have been deposited to the ProteomeXchange Consortium via the PRIDE (Vizcaíno et al., 2016) partner repository with the dataset identifier PXD008017. In addition to the datasets generated in this study, we also re-analyzed the UNEVEN eight 4 h, eight 8 h and four 2D-LC-MS/MS datasets generated by Kleiner et al. (2017). The corresponding raw data has been deposited to PRIDE with the dataset identifier PXD006118.

## Author Contributions

MK conceived the study, obtained and created bacterial stocks for mock communities. MK and TH designed the experiments. TH and R-AH prepared samples for mass spectrometry. TH measured samples, analyzed the data, and wrote the manuscript with input from MK. AK did mass spectrometry measurements. MS and AK revised the manuscript. All authors read and approved the final manuscript.

## Conflict of Interest Statement

The authors declare that the research was conducted in the absence of any commercial or financial relationships that could be construed as a potential conflict of interest.

## Footnotes

1 https://tools.thermofisher.com/content/sfs/manuals/87961-MAN-4820-4103-UltiMate3000-RSLCnano-Applicationsmanual-V1R3.pdf

2 http://thegpm.org/crap/

3 http://bioinformatics.psb.ugent.be/webtools/Venn/

## Abbreviations

1D-LC-MS/MS: one-dimensional liquid chromatography coupled to tandem mass spectrometry
2D-LC-MS/MS: two-dimensional liquid chromatography coupled to tandem mass spectrometry
ABC: ammonium bicarbonate
APS: ammonium persulfate
DTT: dithiothreitol
FASP: filter-aided sample preparation
GeLC-MS/MS: 1D gel electrophoresis coupled to tandem mass spectrometry
HPLC: high performance liquid chromatography
LC: liquid chromatography
MS: mass spectrometry
MS/MS: tandem mass spectrometry
NSAF: normalized spectral abundance factor
pI: isoelectric point
PSM: peptide spectrum match
protein-SIF: protein stable isotope fingerprinting
RP: reversed-phase
SCX: strong cation exchange
SDS: sodium dodecyl sulfate
SIP: stable isotope probing
TEMED: tetramethylethylenediamine
