# Supplementary material for: More Is Not Always Better: Evaluation of 1D and 2D-LC-MS/MS Methods for Metaproteomics
Source: Front Microbiol. 2019 Feb 14;10:238. doi: 10.3389/fmicb.2019.00238 (PMC6383543; doi:10.3389/fmicb.2019.00238)
Supplement: Supplementary file 1 [file Data_Sheet_1.docx]

Supplementary Material

More is not always better: Evaluation of 1D and 2D-LC-MS/MS methods for metaproteomics

Tjorven Hinzke^1,2,3,*^, Angela Kouris^1^, Rebecca-Ayme Hughes^4^, Marc Strous^1^ and Manuel Kleiner^1,4,*^

^1^ Department of Geoscience, University of Calgary, Calgary, Canada

^2^ Institute of Pharmacy, Department of Pharmaceutical Biotechnology, University of Greifswald, Greifswald, Germany

^3^ Institute of Marine Biotechnology e. V., Greifswald, Germany

^4^ Department of Plant & Microbial Biology, North Carolina State University, Raleigh, USA

*** Correspondence:** tjorven.hinzke@stud.uni-greifswald.de, Tel.: +49-3834-420-5949; manuel_kleiner@ncsu.edu, Tel.: +1-919-515-3792

# Contents

| **Supplementary Tables** | **Excel file** |
| --- | --- |
| **Table S1:** Overview of the LC gradients used for comparison of 1D, 2D-LC-MS/MS and GeLC separation methods for metaproteomics. | sheet Table S1 |
| **Table S2:** Overview of the identification metrics for the 1D, 2D-LC-MS/MS and GeLC methods used in this study. For methods that were run in replicates the mean is shown. | sheet Table S2 |
| **Table S3:** Number of identified protein groups, unique peptides and PSMs per organism in the mock community for repeated runs of 1D and 2D-LC-MS/MS methods evaluated in this study. | sheet Table S3 |
| **Table S4:** % NSAF summed for each organism in the mock community for 1D and 2D LC-MS methods measured in four biological replicates and the GeLC method, measured in biological triplicate. | sheet Table S4 |
| **Table S5:** Overlapping protein groups between protein identifications for technical and biological replicates of the mock community, measured with different LC methods. | sheet Table S5 |
| **Table S6:** Number of identified protein groups, unique peptides and PSMs per organism in the mock community for the 1D, 2D-LC-MS/MS and GeLC methods tested in this study | sheet Table S6 |
| **Table S7:** Relative bias of selected LC methods regarding identification of long/short proteins and proteins with low/high pI. | sheet Table S7 |
| **Figure S1.** Overview of results from selected LC methods. | **page 3** |

**Figure S1.** Overview of results from selected LC methods. **(A)** Number of identified protein groups. **(B)** Number of identified unique peptides. **(C)** Number of PSMs. **(D)** Runtime needed for the method. For **(A)**-**(C)**, means are shown. For 1D|8h_50≥2_, the mean of all 1D|8h, 1D|8h_50_2_ and the 1D|8h_50_2.5_ is shown. Numbers above bars show the total identification numbers. Percentages show the proportional increase in method performance.
